# Supplementary material for: Mistargeting of aggregation prone mitochondrial proteins activates a nucleus-mediated posttranscriptional quality control pathway in trypanosomes
Source: Nat Commun. 2022 Jun 2;13:3084. doi: 10.1038/s41467-022-30748-z (PMC9163028; doi:10.1038/s41467-022-30748-z)
Supplement: Supplementary file 1 — Supplementary Information [file 41467_2022_30748_MOESM1_ESM.pdf]

SUPPLEMENTARY INFORMATION

Mistargeting of aggregation prone mitochondrial proteins activates a nucleus-mediated posttranscriptional quality control pathway in trypanosomes

Caroline E. Dewar[1)], Silke Oeljeklaus[2), 3)], Jan Mani[1)], Wignand W. D. Mühlhäuser[3)], Corinne von Känel[1)], Johannes Zimmermann[2)], Torsten Ochsenreiter[4)], Bettina Warscheid[2), 3), 5)*] and André Schneider[1) *]

[1)] Department of Chemistry, Biochemistry and Pharmaceutical Sciences, University of Bern, Freiestrasse 3, Bern CH-3012, Switzerland

[2)] Faculty of Chemistry and Pharmacy, Department of Biochemistry, Theodor Boveri-Institute, University of Würzburg, 97074 Würzburg, Germany

[3)] Biochemistry and Functional Proteomics, Institute of Biology II, Faculty of Biology, University of Freiburg, 79104 Freiburg, Germany

[4)] Institute of Cell Biology, University of Bern, Baltzerstrasse 4, Bern CH-3012, Switzerland

[5)] CIBSS Centre for Integrative Biological Signalling Studies, University of Freiburg, 79104 Freiburg, Germany

* Corresponding authors:

Bettina.Warscheid@biologie.uni-freiburg.de, andre.schneider@dcb.unibe.ch

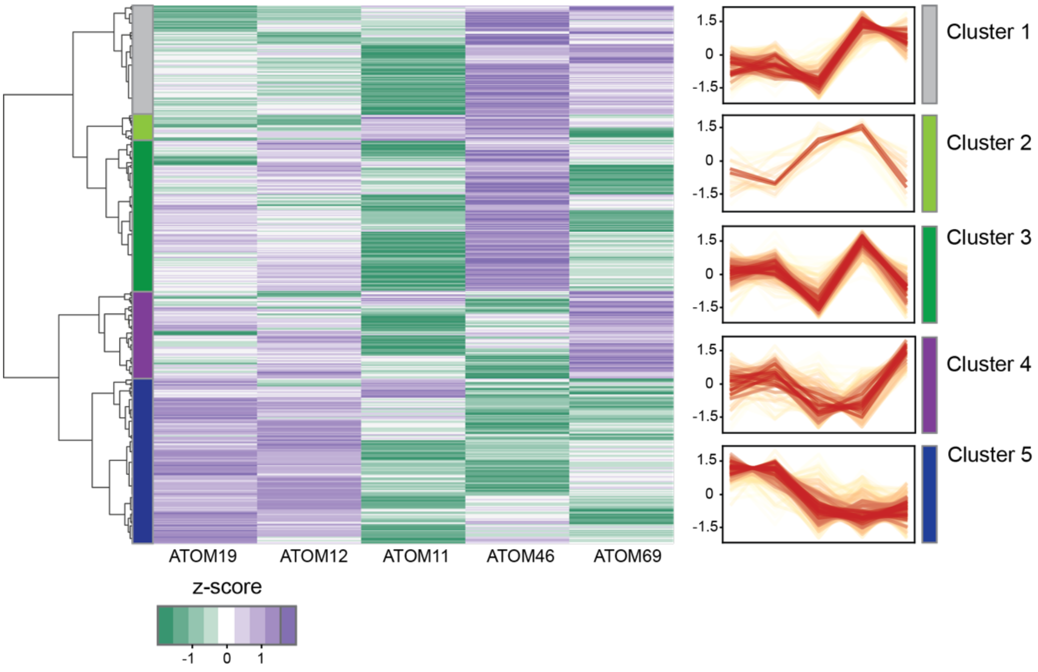

Cluster 1; n=96
OXPHOS: ATPTB14; #1
MITORIBO: mS50, mS52; #2

Cluster 2; n=23
OXPHOS: SDH1, SDH2C, putative complex I subunit Tb927.7.7330; #3
MITORIBO: mt-IF-3; #1

Cluster 3; n=132
OXPHOS: NDUFS1, putative complex I subunit Tb927.10.7610, SDH3, SDH7, SDH9, putative complex III subunit Tb927.11.162200, ATPTB3, ATPF1G, ATPTB8/*e*/ATPEG2, ATPTB9/*g*/ATPEG1, F1 ATP synthase subunit delta, F1 ATP synthase subunit epsilon, P18; #13
MITORIBO: mL64, mL92, mS34, mS35, mS47, uS10m; #6

Cluster 4; n=77
OXPHOS: NDUFA5, putative complex I subunit Tb927.8.5560; #2
MITORIBO: mS26, mS49, mS54, mS58, mS67; #5

Cluster 5; n=145
OXPHOS: NDUFA2, NDUFA6, NDUFV2, putative complex I subunits Tb927.3.3660, Tb927.6.1410 and Tb927.9.11660, SDH2N, SDH5, SDH6, SDH10, COXIV, COXV, COXVI, COXVII, COXIX, COXX, ATP5/OSCP, ATPTB1, ATPTB10/*i*/*j*, ATPTB11, ATPTB12, ATPTB13/8, ATPTB2/*d*, ATPTB3, ATPTB4, ATPTB5/*f*, ATPTB6, ATPTB7/*k*; #28
MITORIBO: bL17m, bL20m, bL21m, bL28m, bL35m, bL9m, bS18m, mL38, mL40, mL41, mL42, mL43, mL49, mL52, mL63, mL67, mL68, mL69, mL73, mL76, mL83, mL84, mL85, mL86, mL89, mS22, mS23, mS33, mS48, mS51, mS55, mS56, mS57, mS62, mS64, mS65, mS69, mS91, uL15m, uL16m, uL23m, uL29m, uL30m, uS11m; #44

Supplementary Figure 1. Clustering of mitochondrial proteins with altered abundance in mitochondrial fractions following RNAi-mediated ablation of different ATOM complex subunits in *T. brucei*.
For the hierarchical clustering, SILAC-MS data were filtered for mitochondrial proteins with a p-value of ≤ 0.01 (determined using a two-sided limma test; not adjusted) following depletion of at least one of the three ATOM core subunits ATOM19, ATOM12, ATOM11 or the two receptor subunits ATOM46 and ATOM69. Due to the overall stronger effect on the downregulation of mitochondrial proteins or ATOM complex stability, SILAC-MS data from RNAi experiments targeting ATOM14 and ATOM40 were not included in the analysis. A Pearson's correlation-based distance matrix was used for the hierarchical clustering using the Ward's method. For visualization of data in a heat map, row-wise z-score transformation was performed. Clustering resulted in 5 clusters as indicated by the colour code. Mitochondrial proteins affected by ATOM69-RNAi are found in cluster 2, 3 and 5. ATOM46-RNAi affected mitochondrial proteins are in cluster 4 and 5. Since depletion of ATOM11 leads to a considerably reduction in the levels of ATOM46 and ATOM69, it is plausible that mitochondrial proteins in clusters 3, 4 and 5 are also reduced in ATOM11-RNAi experiments. Proteins of OXPHOS complexes and mitochondrial ribosomes (MITORIBO) are predominantly present in cluster 3 and 5. OXPHOS, oxidative phosphorylation system; n, total number of proteins in each cluster; #, number of OXPHOS subunits or mitochondrial ribosomes present in cluster 1-5. Proteins are listed according to their gene name.

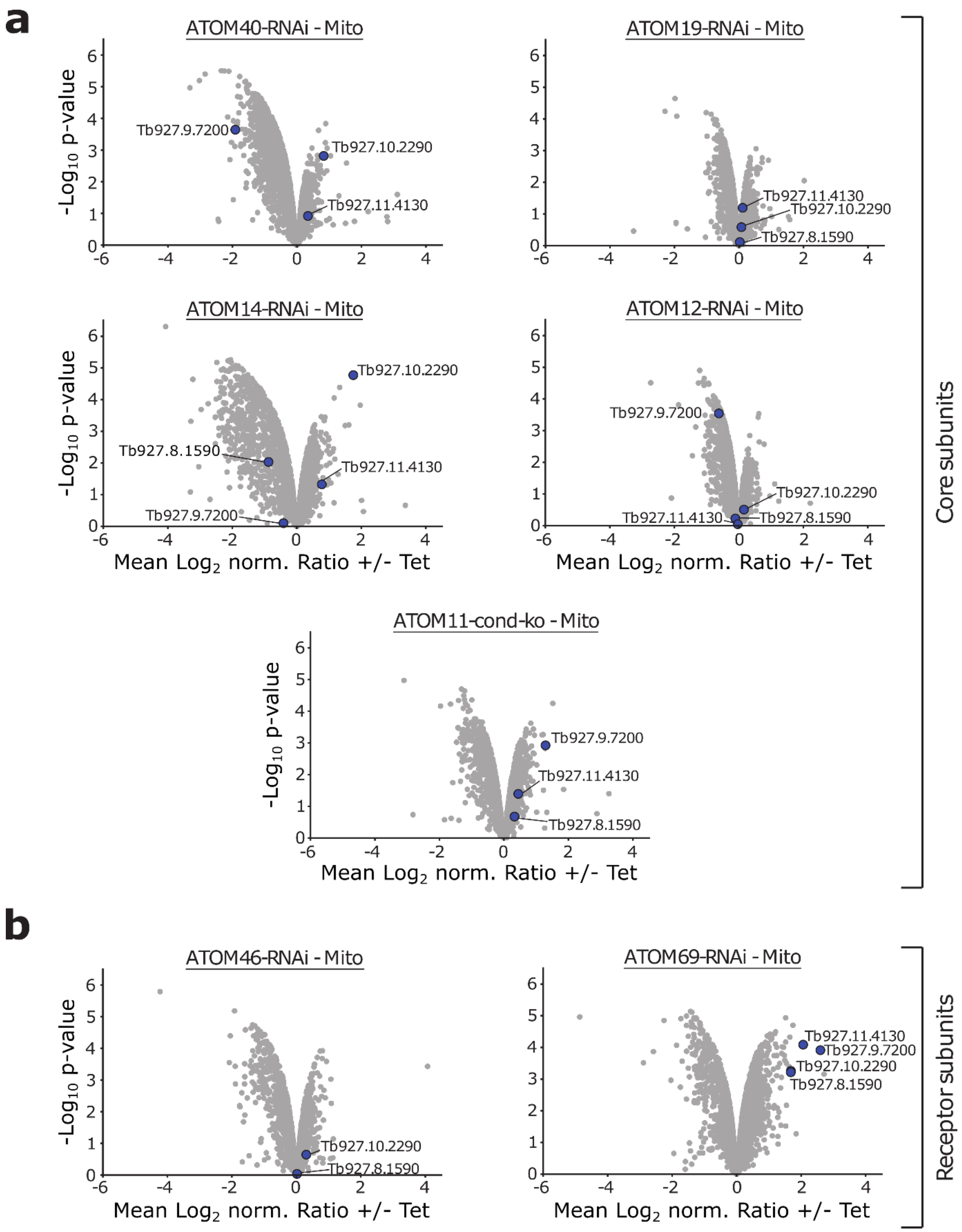

Supplementary Figure 2. Effect of the ablation of ATOM complex subunits on the abundance of candidate proteins implicated in the degradation of mislocalised proteins. a, **b** Same plots as shown in Figures 1b and 1c highlighting the four non-mitochondrial proteins with a potential role in mitochondrial quality control in datasets acquired for the atypical translocase of the outer membrane (ATOM) core subunits ATOM40, ATOM19, ATOM14, ATOM12 and ATOM11, a,and the ATOM receptor subunits ATOM46 and ATOM69, b. All other proteins detected are depicted in grey.

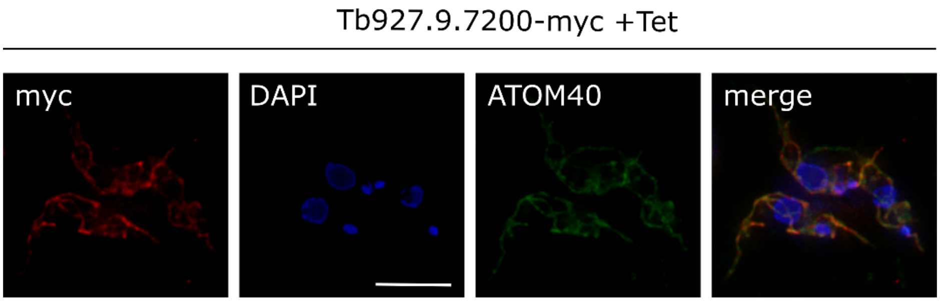

Supplementary Figure 3. Tb927.9.7200-myc localises to the mitochondrion. Immunofluorescence analysis of a cell line allowing tetracycline-inducible expression of myc tagged Tb927.9.7200. Atypical translocase of the outer membrane 40 (ATOM40) serves as a mitochondrial marker. Scale bar 10 µm. Cells were induced for 3d. This IF analysis was repeated independently twice with similar results.

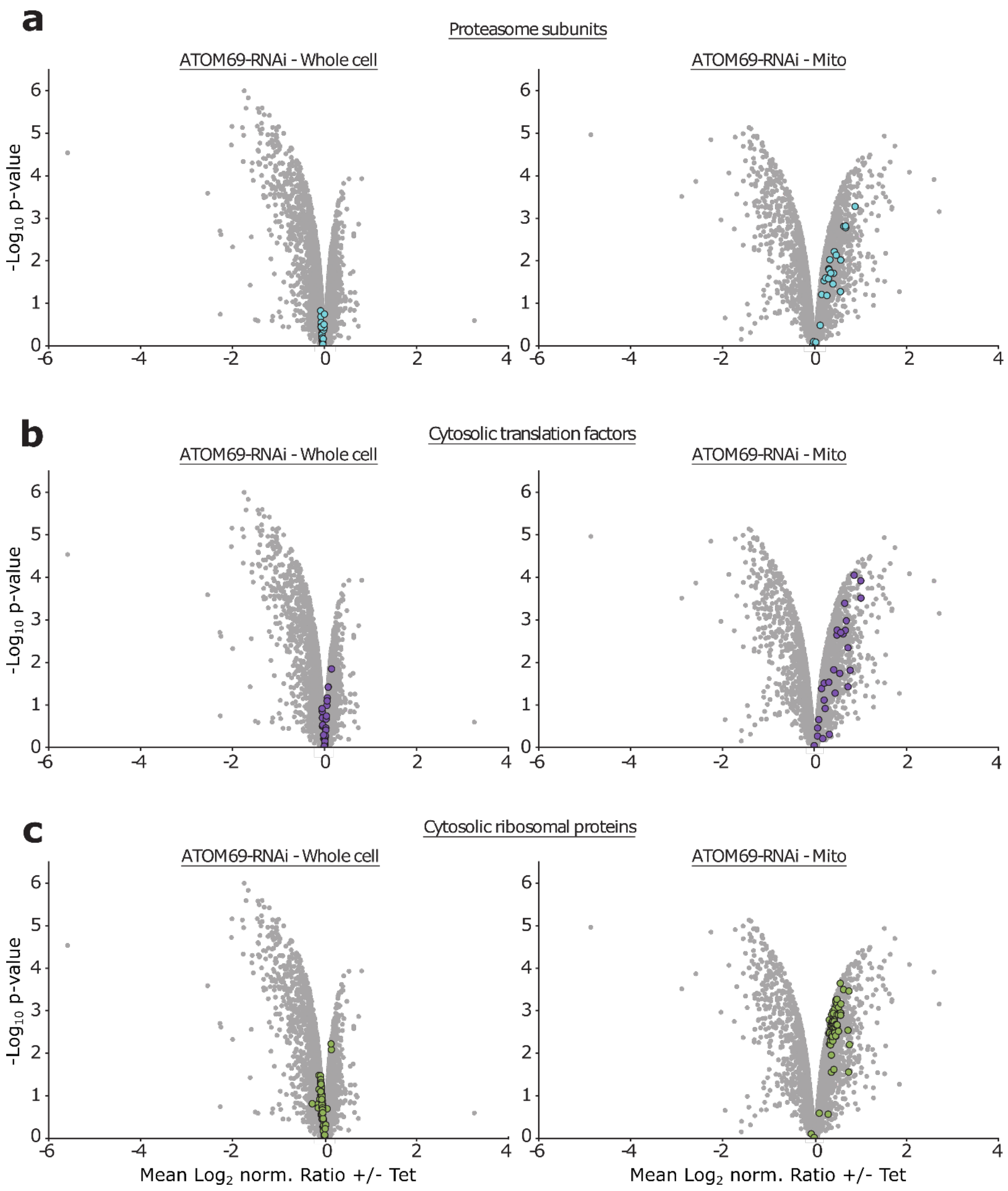

Supplementary Figure 4. Ablation of ATOM69 stimulates mitochondrial recruitment of the proteasome, cytosolic translation factors and cytosolic ribosomal proteins. a-c Visualisation of stable isotope labelling by amino acids in cell culture (SILAC)-based quantitative mass spectrometry (MS) data of whole cell (left panels) and crude mitochondrial (Mito) extracts (right panels) obtained in ATOM69 RNAi experiments. Proteasomal subunits, cytosolic translation factors, and cytosolic ribosomal proteins are highlighted in light blue, a, purple, b, and green, c, respectively. All other proteins detected are depicted in grey.

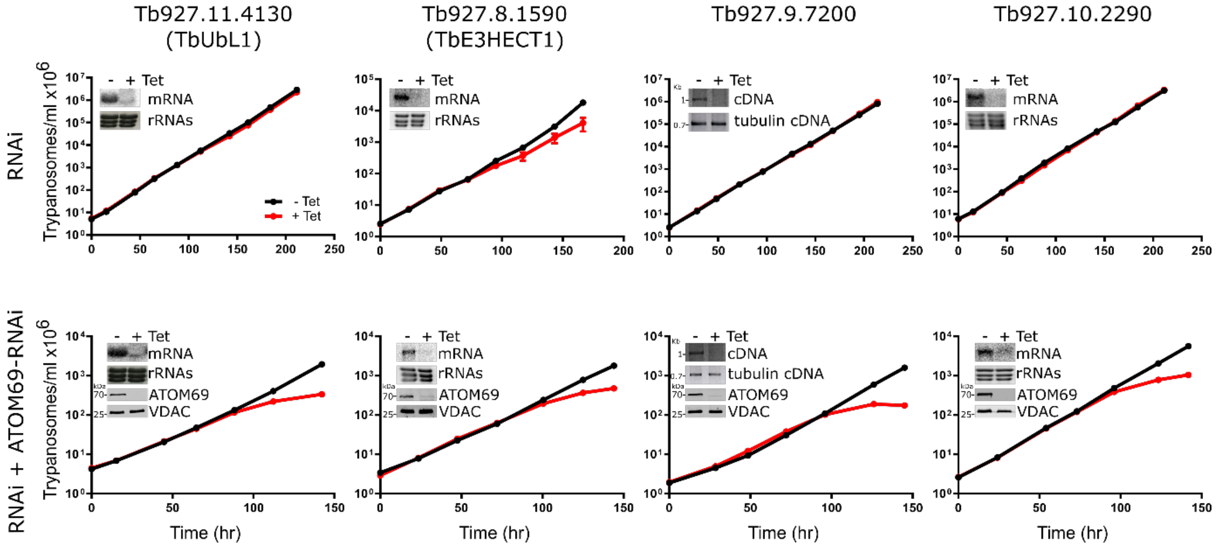

Supplementary Figure 5. Verification and growth of single and double RNAi cell lines. Growth curves of uninduced (-Tet) and induced (+Tet) single and double RNAi cell lines as indicated. Data are presented as mean values with error bars corresponding to the standard deviation (n=3). Insets show the efficiency of RNAi for the indicated proteins two days after tetracycline induction, as analysed by northern blots (Tb927.11.4130, Tb927.8.1590, Tb927.10.2290), RT PCR (Tb927.9.7200) or immunoblots (ATOM69). Cytosolic rRNAs stained with ethidium bromide (EtBr), tubulin mRNA-derived cDNA or voltage dependent anion channel (VDAC) serve as loading controls. Source data are provided as a Source Data file.

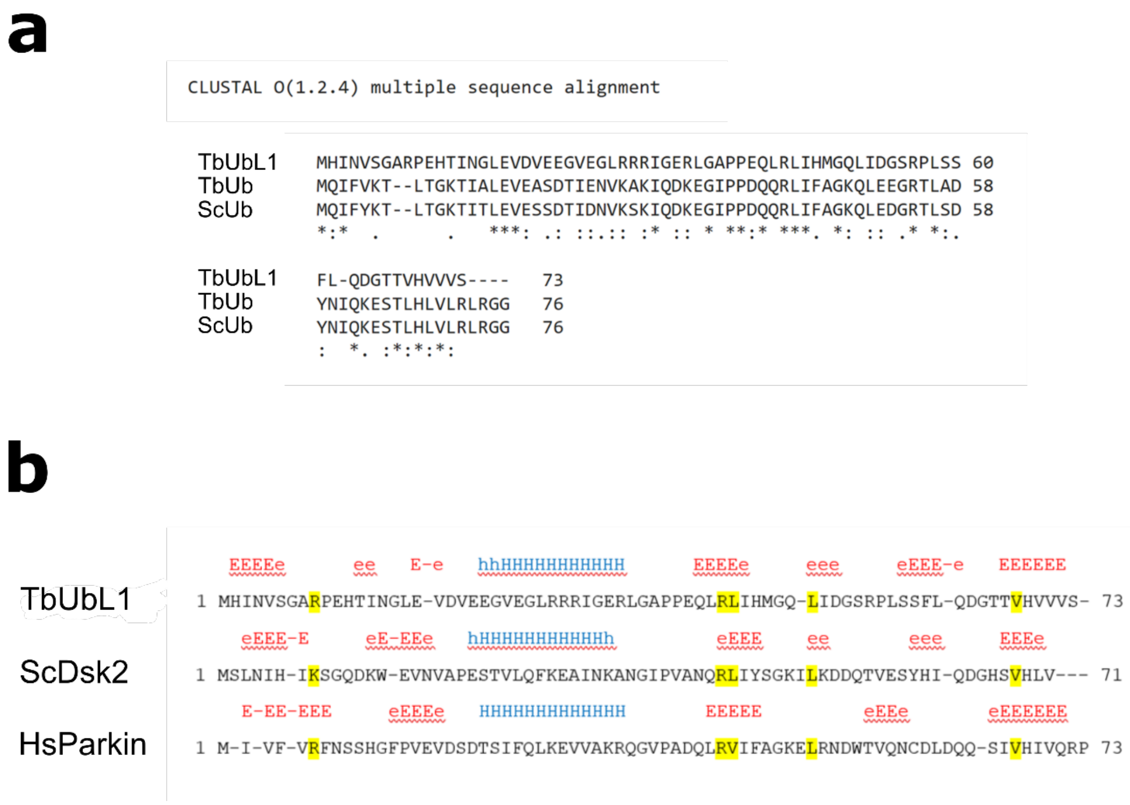

Supplementary Figure 6. The ubiquitin-like domain of TbUbL1 has little sequence homology to ubiquitin but contains the typical beta-grasp fold of ubiquitin. a Sequence alignment of predicted ubiquitin-like fold domain of ubiquitin-like protein 1 (TbUbL1) with monoubiquitin of *T. brucei* (TbUb, Tb927.11.9920) and *S. cerevisiae* (ScUb) by Clustal Omega [1]. **b** Comparison of the ubiquitin-like domains of TbUbL1 with the ubiquitin-like domains of Dsk2 from *S. cerevisiae* (ScDsk2), and parkin from *H. sapiens* (HsParkin). Predicted secondary structures of the first 71 or 73 amino acids of these proteins as determined by the PHD programme are shown [2]. E represents a β strand, and H represents an α helix (upper case indicates a prediction of >82%). PIM motif amino acids are highlighted in yellow.

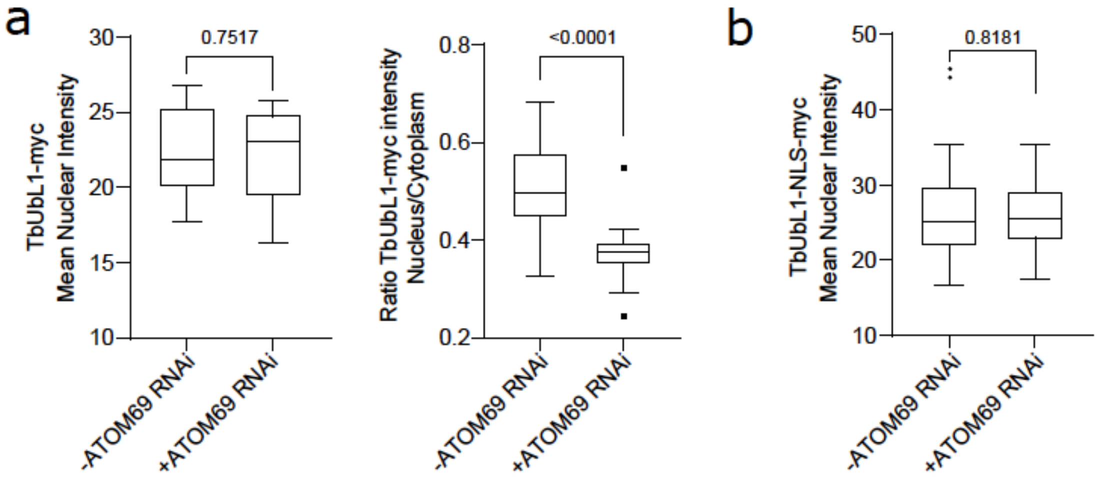

Supplementary Figure 7. Quantification of immunofluorescence (IF) analyses. **a**, left panel, box-and-whisker plot of nuclear ubiquitin-like protein 1 (TbUbL1)-myc signals of the IF analyses shown in main Fig. 4a (n=10 each). Right panel, box-and-whisker plot of the ratio between the nuclear and cytoplasmic TbUbL1-myc signals of the IF analyses shown in main Fig. 4a (n=20 each). **b**, box-and-whisker plot of the nuclear TbUbL1-myc signals of the two cell lines shown in Fig. 7a (-ATOM69 RNAi: n=30; +ATOM69 RNAi n=20). For both panels, the box extends from the 25th to 75th percentiles, the median is indicated. Upper and lower whisker 75th and 25th percentile plus/minus 1.5 times inter quartile range (IQR), respectively. Value less or more than the sum of the 25th or 75th percentile plus/minus 1.5 IQR are plotted as individual points. The *p* values indicated in the plots were calculated via two-tailed unpaired *t* tests.

Images used for analysis originated from a single stack. Uninduced and induced cell lines were blinded prior to analysis. Only cells in focus were used for measurements. Mean nuclear intensity measurements as shown in a and b were done using a circular fixed size region of interest (ROI) and the measure function in ImageJ2 (2.3.0). Background was subtracted using identical ROI in proximity of the cell. For the ratio between nucleus and cytoplasm we measured total intensity of the cell body area and nuclear area using the ROI tool and measure function. In both cases the background of the identical ROI that was measured in proximity to the cell, was subtracted. We subtracted the value of the nucleus from the total cell body to generate the cytoplasmic value. From cytoplasmic and nuclear intensities, we generated the ratios shown in a. Source data are provided as a Source Data file.

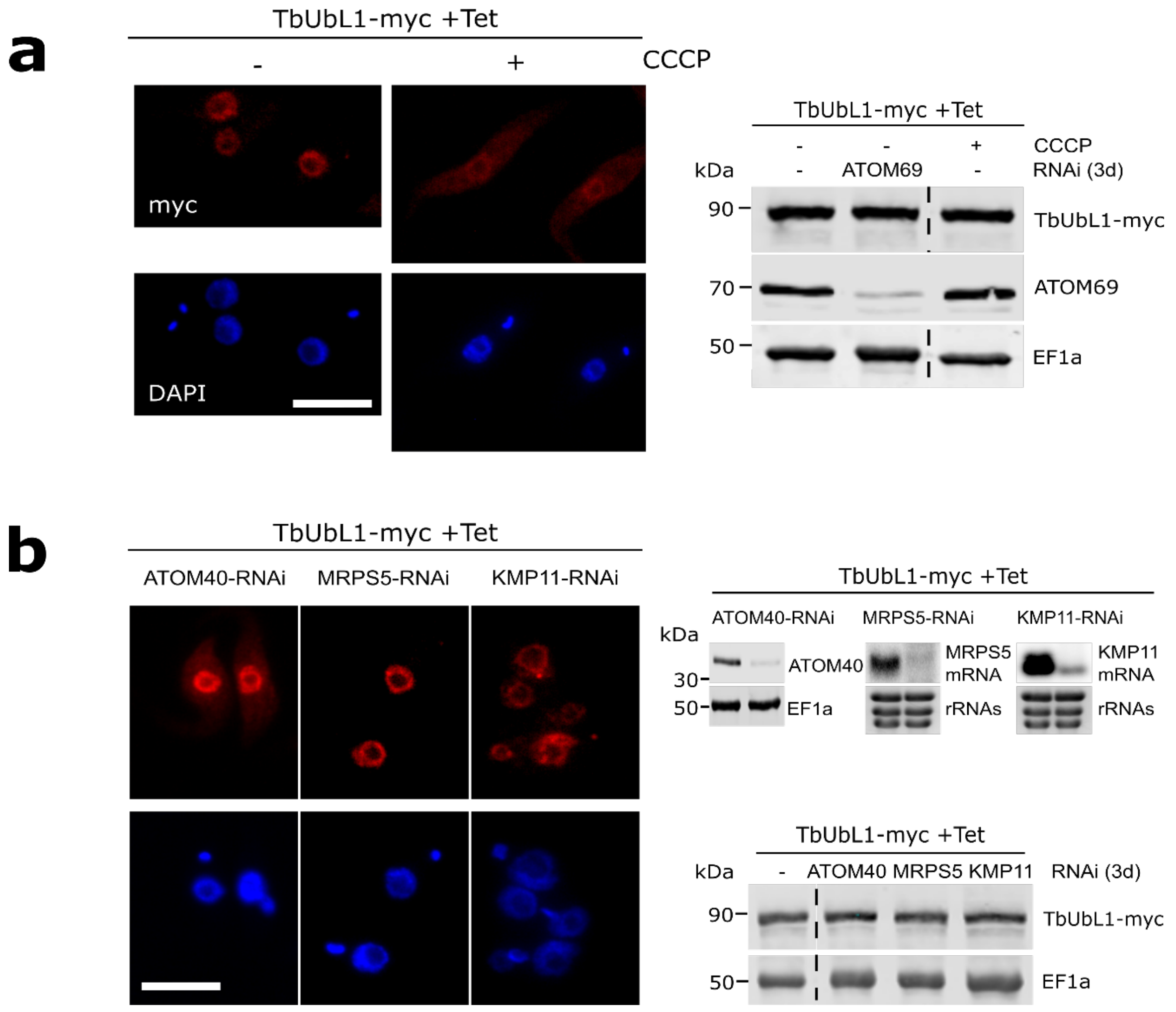

Supplementary Figure 8. Different triggers of the nuclear release of TbUbL1. a Left panels, immunofluorescence (IF) analysis of cells expressing ubiquitin-like protein 1 (TbUbL1-myc), untreated and treated with 10 µM carbonylcyanide-m-chlorphenylhydrazone (CCCP) for 1d. Scale bar 10 µm. This IF analysis was repeated independently twice with similar results. Right panels, levels of TbUbL1-myc expressed in the indicated cell lines. Immunoblots were probed with anti-myc-tag and atypical translocase of the outer membrane 69 (ATOM69) antibodies. Elongation factor 1a (EF1a) is used as a loading control. **b** Left panels, IF analyses of induced RNAi cell lines targeting ATOM40, the mitoribosomal protein S5 (MRPS5) and the cytokinesis factor KMP11 that simultaneously express TbUbL1-myc. This IF analysis was repeated independently twice with similar results. Right top panels, the efficiency of RNAi for the indicated proteins two days after tetracycline induction, as analysed by northern blots (MRPS5, KMP11) or immunoblots (ATOM40). Cytosolic rRNAs or EF1a serve as loading controls. Right bottom panels, levels of TbUbL1-myc in the cell lines shown in the top panel. EF1a is used as a loading control. For all gels, $2x10^6$ cells were loaded per lane. Black dotted line indicates that samples were run on the same gel, but lanes were non-adjacent.

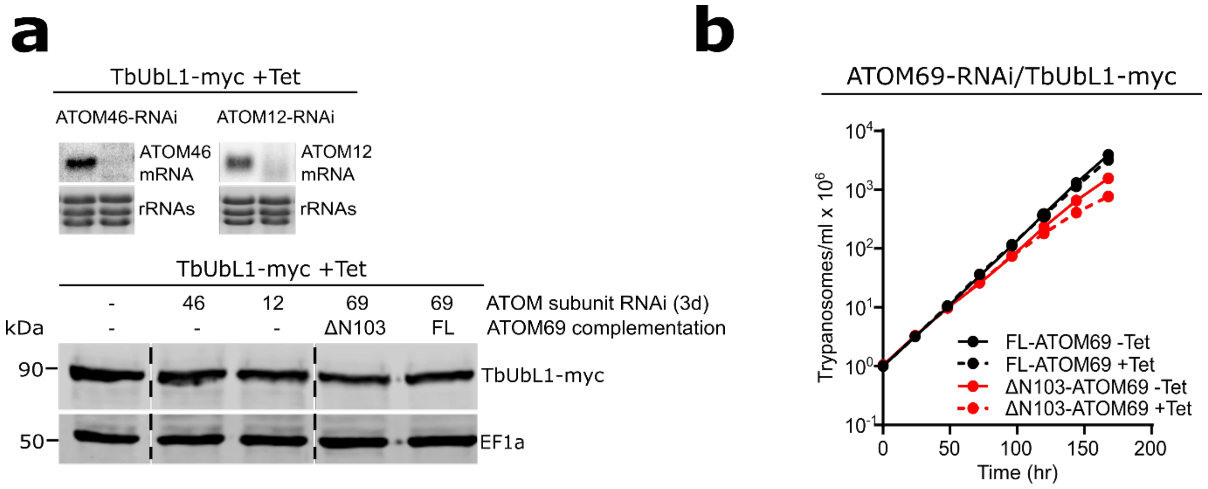

Supplementary Figure 9. a Top panel, the efficiency of atypical outer membrane translocase 46 (ATOM46) or ATOM12 RNAi two days after tetracycline induction, as analysed by northern blots. Bottom panel, levels of ubiquitin-like protein 1 (TbUbL1-myc) in the indicated cell lines. Elongation factor 1a (EF1a) is used as a loading control. For all gels, $2x10^6$ cells were loaded per lane. Black dotted line indicates that samples were run on the same gel, but lanes were non-adjacent. **b** Growth curve of the ATOM69-RNAi/ΔN103-ATOM69/TbUbL1-myc and the ATOM69/FL-ATOM69/TbUbL1-myc cell line as indicated, uninduced (-Tet) or induced (+Tet). Data are presented as mean values with error bars corresponding to the standard deviation (n=3). Source data are provided as a Source Data file.

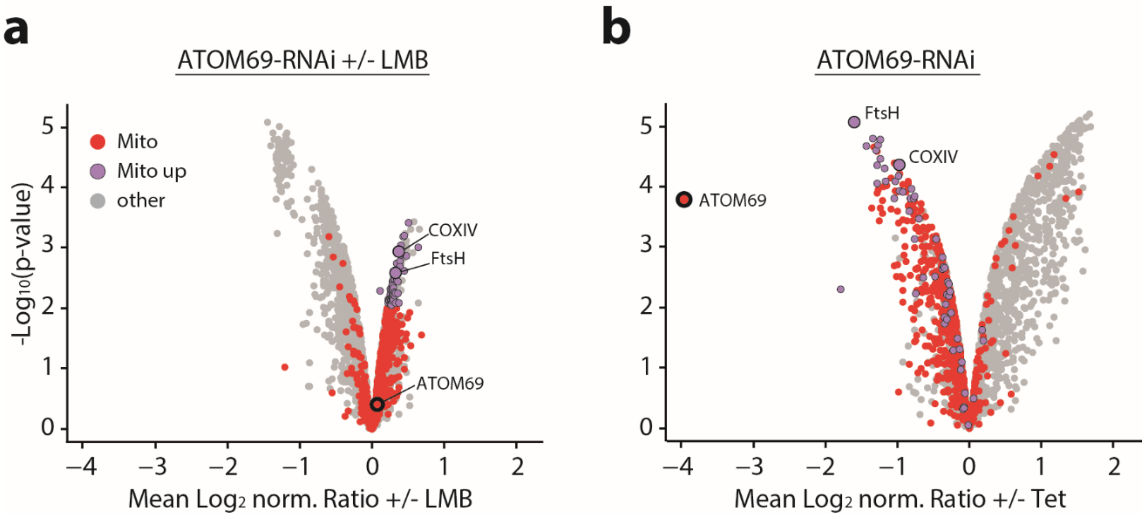

Supplementary Fig. 10. Mitochondrial proteome changes after ablation of ATOM69 in the presence and absence of leptomycin B (LMB). a mitochondria-enriched extracts of induced atypical translocase of the outer membrane 69 (ATOM69)-RNAi cells that were incubated with 50 ng/ml LMB or left untreated were subjected to stable isotope labelling by amino acids in cell culture (SILAC)-based quantitative mass spectrometry (MS). The volcano plot depicts mitochondrial proteins (red dots) as defined in [3]. Mitochondrial protein that are significantly enriched after LMB treatment (Mito up) are indicate in purple. The model substrates FtsH and cytochrome oxidase subunit IV (COXIV) are highlighted. **b** Volcano plot of a control experiment showing mitochondria-enriched extracts of uninduced and induced ATOM69-RNAi cells that were subjected to SILAC-based quantitative mass spectrometry (MS). Colours as in a. Triplicate experiments were analysed.

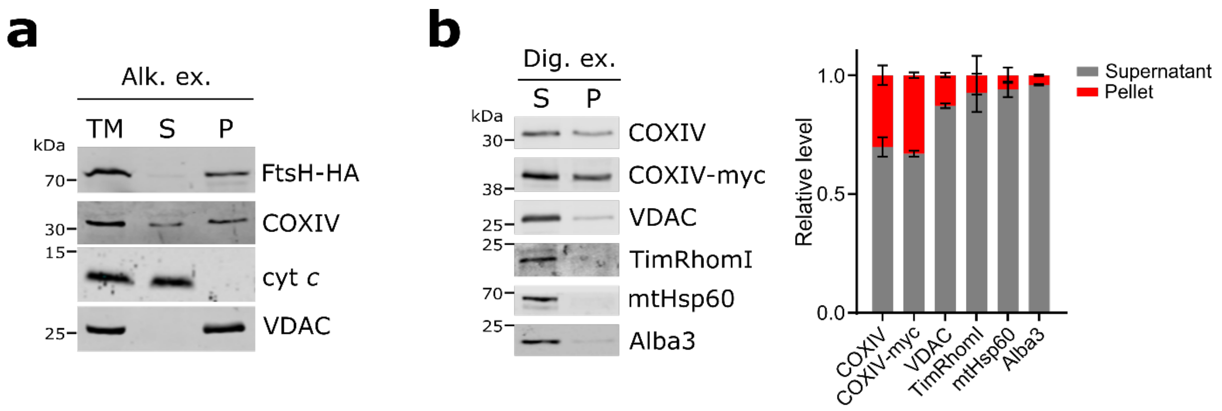

Supplementary Figure 11. Cytochrome oxidase subunit IV (COXIV) has a tendency for aggregation. a A digitonin-extracted mitochondria-enriched fraction (TM) from a cell line expressing in situ tagged FtsH-HA was subjected to alkaline carbonate extraction performed at pH 11.5 resulting in membrane-enriched pellet (P) and soluble supernatant (S) fractions. Subsequent immunoblots were decorated with anti-HA or anti-COXIV and antisera against voltage dependent anion channel (VDAC) and cytochrome *c*, which serve as markers for integral membrane and soluble proteins, respectively. $5x10^6$ cell equivalents were loaded per lane. **b** Left panel, immunoblot analysis of digitonin-extracted mitochondria-enriched pelleted (P) and soluble cytosolic (S) fractions of cells expressing COXIV-myc. The immunoblots were probed with anti-myc and antisera against COXIV, VDAC (OM), TimRhomI (IM), mitochondrial heat shock protein 60 (mtHsp60) (soluble, matrix) or Alba3 (soluble, cytosol) respectively. $2x10^6$ cell equivalents were loaded per lane. Right panel, quantifications of these protein levels from triplicate immunoblots. Data are presented as mean values with error bars corresponding to the standard deviation of the mean of three independent biological replicates. Source data are provided as a Source Data file.

References

1. Sievers F*, et al.* Fast, scalable generation of high-quality protein multiple sequence alignments using Clustal Omega. *Mol Syst Biol* **11**, 539 (2011).

2. Rost B, Sander C. Prediction of protein secondary structure at better than 70% accuracy. *Journal of molecular biology* **232**, 584-599 (1993).

3. Peikert CD*, et al.* Charting Organellar Importomes by Quantitative Mass Spectrometry. *Nat Commun* **8**, 15272 (2017).

Full scans for Supplementary Figure 5

1 -tet

2 +tet

Tb927.11.4130:

RNAi (left), RNAi + ATOM69-RNAi (right)

1 2   1 2

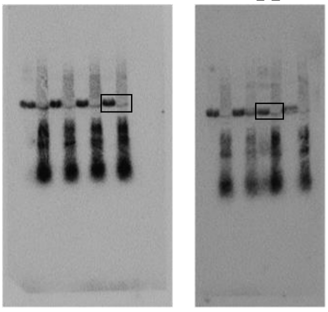

mRNA

1 2   1 2

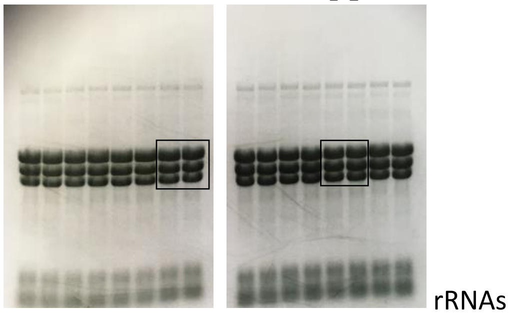

rRNAs

1 2

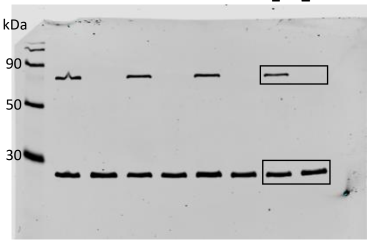

ATOM69/VDAC

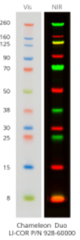

Tb927. 8.1590:

RNAi (top), RNAi + ATOM69-RNAi (bottom)

1 2

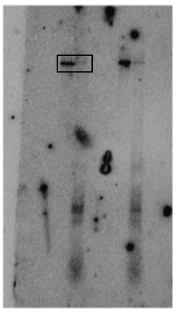

mRNA

1 2

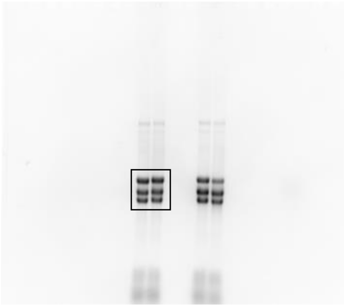

rRNAs

1 2

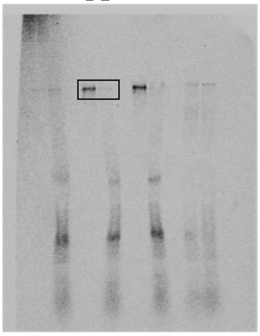

mRNA

1 2

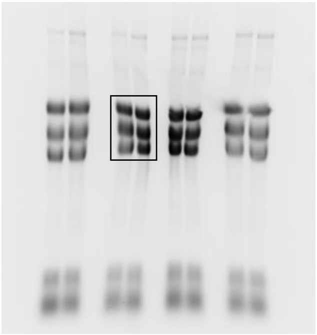

rRNAs

1 2

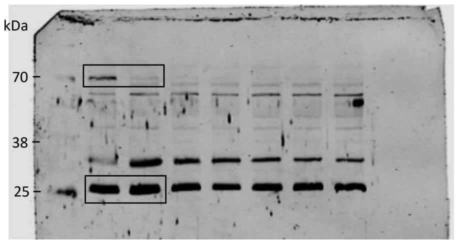

ATOM69/VDAC

Tb927.9.7200:

RNAi (left), RNAi + ATOM69-RNAi (right)

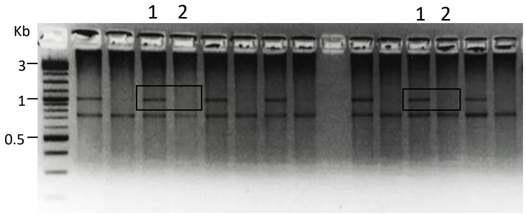

cDNA

RNAi (top), RNAi + ATOM69-RNAi (bottom)

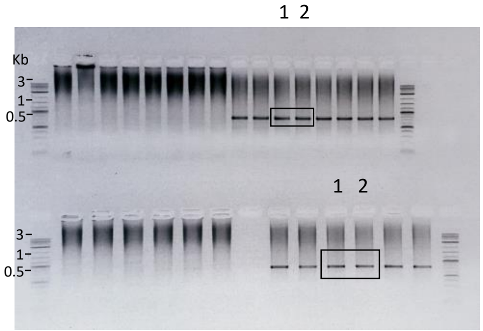

tubulin cDNA

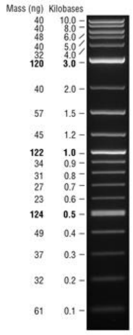

2-log ladder (NEB)

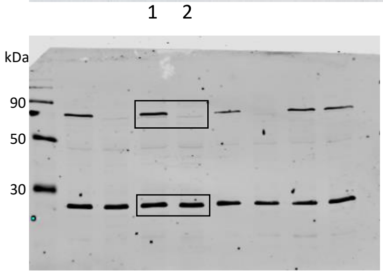

ATOM69/VDAC

Tb927.10.2290:

RNAi (left two), RNAi + ATOM69-RNAi (right two)

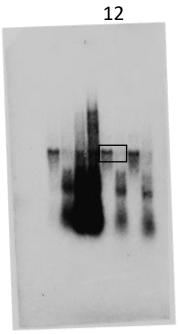

mRNA

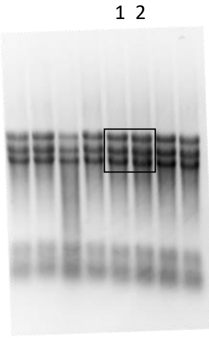

rRNAs

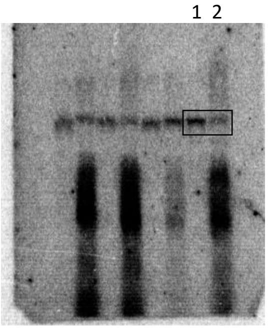

mRNA

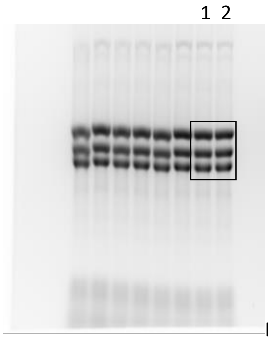

rRNAs

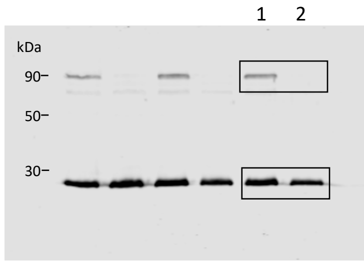

ATOM69/VDAC

Full scans for Supplementary Figure 8a

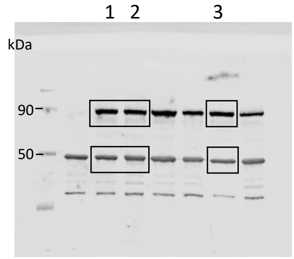

myc/EF1a

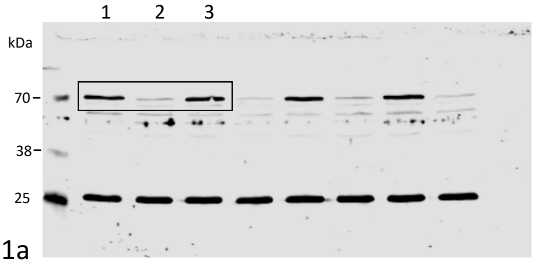

ATOM69

1 -tet ATOM69 RNAi

2 +tet ATOM69 RNAi

3 +CCCP

Full scans for Supplementary Figure 8b

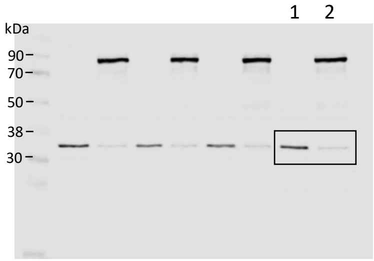

ATOM40

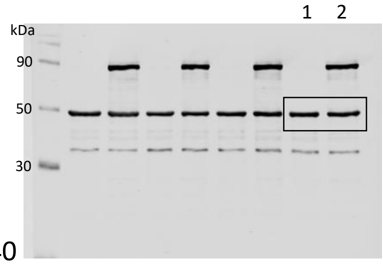

EF1a

1 -tet

2 +tet

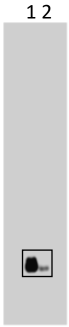

KMP11 mRNA

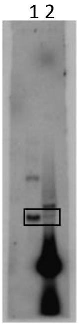

MRPS5 mRNA

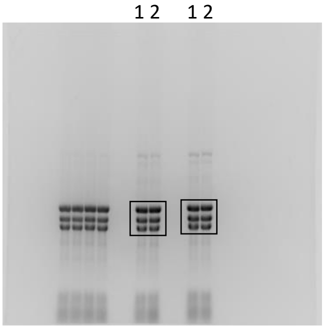

rRNAs

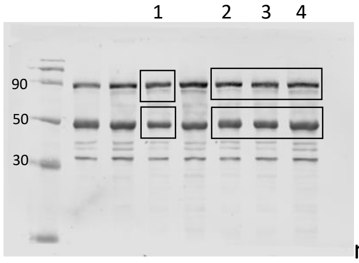

myc/EF1a

1 -tet

2 +tet ATOM40 RNAi

3 +tet MRPS5 RNAi

4 +tet KMP11 RNAi

Full scans for Supplementary Figure 9a

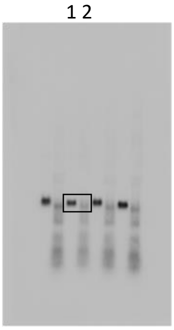

ATOM12 mRNA

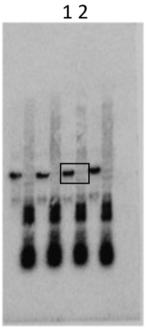

ATOM46 mRNA

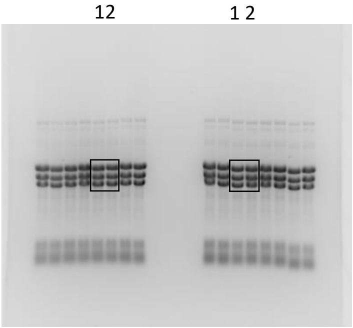

rRNAs

1 -tet

2 +tet

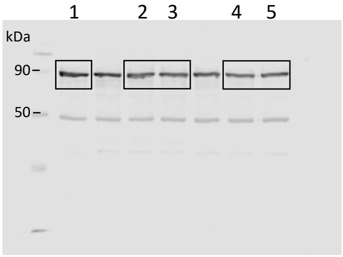

myc

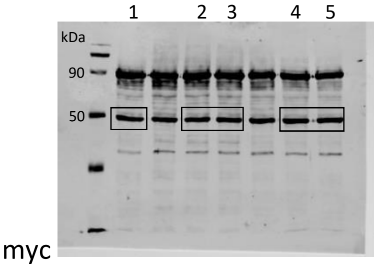

EF1a

1 -tet

2 +tet ATOM46 RNAi

3 +tet ATOM12 RNAi

4 +tet ATOM69 RNAi + ΔN103-ATOM69

5 +tet ATOM69 RNAi + FL-ATOM69

Full scans for Supplementary Figure 11a

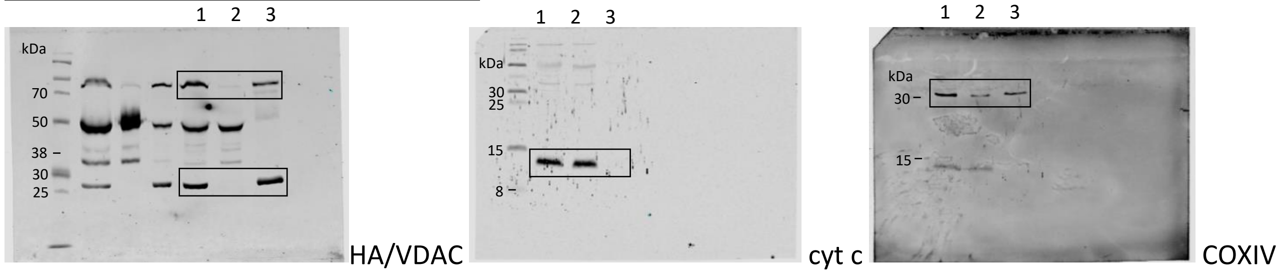

1 mitochondria-enriched fraction

2 supernatant

3 pellet

Full scans for Supplementary Figure 11b

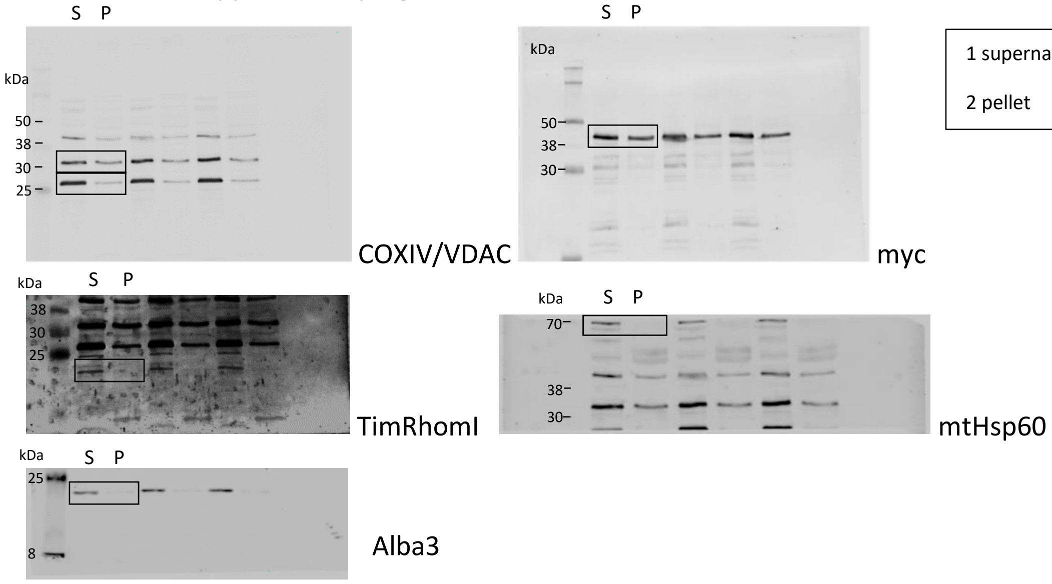

1 supernatant

2 pellet
