# Supplementary material for: Mistargeting of aggregation prone mitochondrial proteins activates a nucleus-mediated posttranscriptional quality control pathway in trypanosomes
Source: Nat Commun. 2022 Jun 2;13:3084. doi: 10.1038/s41467-022-30748-z (PMC9163028; doi:10.1038/s41467-022-30748-z)
Supplement: Supplementary file 3 — Description of Additional Supplementary Information [file 41467_2022_30748_MOESM3_ESM.docx]

**Description of Additional Supplementary Files**

Name: Supplementary information
Description: Supplementary Figures 1-11; full scans supplementary Fig. 5, 8ab, 9a and 11ab

Name: Supplementary data 1

Description: Supplementary data for Fig. 1ab

Name: Supplementary data 2

Description: Supplementary data for Fig. 5ab

Name: Supplementary data 3

Description: Supplementary data for supplementaty Fig. 10ab
