# Supplementary material for: Mistargeting of aggregation prone mitochondrial proteins activates a nucleus-mediated posttranscriptional quality control pathway in trypanosomes
Source: Nat Commun. 2022 Jun 2;13:3084. doi: 10.1038/s41467-022-30748-z (PMC9163028; doi:10.1038/s41467-022-30748-z)
Supplement: Supplementary file 7 — Reporting Summary [file 41467_2022_30748_MOESM7_ESM.pdf]

nature portfolio

Corresponding author(s): Corresponding authors: André Schneider, Bettina Warscheid NCOMMS-21-31553

Last updated by author(s): 9. February 22

Reporting Summary

Nature Portfolio wishes to improve the reproducibility of the work that we publish. This form provides structure for consistency and transparency in reporting. For further information on Nature Portfolio policies, see our Editorial Policies and the Editorial Policy Checklist.

Statistics

For all statistical analyses, confirm that the following items are present in the figure legend, table legend, main text, or Methods section.

n/a	Confirmed	
☐	☒	The exact sample size (*n*) for each experimental group/condition, given as a discrete number and unit of measurement
☐	☒	A statement on whether measurements were taken from distinct samples or whether the same sample was measured repeatedly
☐	☒	The statistical test(s) used AND whether they are one- or two-sided<br>*Only common tests should be described solely by name; describe more complex techniques in the Methods section.*
☒	☐	A description of all covariates tested
☐	☒	A description of any assumptions or corrections, such as tests of normality and adjustment for multiple comparisons
☐	☒	A full description of the statistical parameters including central tendency (e.g. means) or other basic estimates (e.g. regression coefficient) AND variation (e.g. standard deviation) or associated estimates of uncertainty (e.g. confidence intervals)
☐	☒	For null hypothesis testing, the test statistic (e.g. *F*, *t*, *r*) with confidence intervals, effect sizes, degrees of freedom and *P* value noted<br>*Give P values as exact values whenever suitable.*
☒	☐	For Bayesian analysis, information on the choice of priors and Markov chain Monte Carlo settings
☐	☒	For hierarchical and complex designs, identification of the appropriate level for tests and full reporting of outcomes
☐	☒	Estimates of effect sizes (e.g. Cohen's *d*, Pearson's *r*), indicating how they were calculated

Our web collection on statistics for biologists contains articles on many of the points above.

Software and code

Policy information about availability of computer code

Data collection	For visualising western blot Licor Odyssey with software version 2.1.15 was used.<br>Quantification of Western blots was performed using Image Studio version 5.2.5.<br>Microscope software: Leica LAS X product version 3.6.20104.0.<br>Microscopic images were analysed using ImageJ version 2.10./1.53c; Java 1.8.0_172 [64-bit] (NIH)
Data analysis	Proteomics: the software package MaxQuant with its integrated search engine Andromeda was used for protein identification and SILAC-based relative quantification (versions 2.0.2.0 for ATOM69 RNAi +/- LMB data, 1.6.10.43 for all other RNAi data, and 1.5.5.1 for IP data)<br>R packages: RankProd (v. 3.11) (https://bioconductor.org/packages/release/bioc/html/RankProd.html) and Limma-Test (https://bioconductor.org/packages/release/bioc/html/limma.html)<br>Python packages: pandas (version 1.2.1; https://pandas.pydata.org/), numpy (v. 1.19.2; https://numpy.org/), seaborn (v. 0.11.1; https://seaborn.pydata.org/), matplotlib (v. 3.3.2; https://matplotlib.org/), scipy (v. 1.5.2; https://www.scipy.org/), scikit-learn (v. 0.24.1; https://scikit-learn.org/stable/)

For manuscripts utilizing custom algorithms or software that are central to the research but not yet described in published literature, software must be made available to editors and reviewers. We strongly encourage code deposition in a community repository (e.g. GitHub). See the Nature Portfolio guidelines for submitting code & software for further information.

Data

Policy information about availability of data

All manuscripts must include a data availability statement. This statement should provide the following information, where applicable:

- Accession codes, unique identifiers, or web links for publicly available datasets
- A description of any restrictions on data availability
- For clinical datasets or third party data, please ensure that the statement adheres to our policy

Mass spectrometric raw data were searched against a fasta file containing all protein sequences for T. brucei TREU927 as provided by the TriTrypDB (version 8.1; https://tritrypdb.org/)

The mass spectrometric data have been deposited to the ProteomeXchange Consortium via the PRIDE partner repository and are available with the identifiers PXD027739 (ATOM subunits RNAi data; Supplementary Data 1a), PXD027652 (TbUbL1-myc IP data; Supplementary Data 2), and PXD031888 (ATOM69 RNAi +/- LMB data; Supplementary Data 3).

To access our proteomics data at the ProteomeXchange Consortium (http://proteomecentral.proteomexchange.org), please use the following usernames and passwords to log into the reviewer accounts:

Project Name: Release of a nuclear ubiquitin-like protein activates proteasomal degradation of mislocalized hydrophobic mitochondrial proteins in trypanosomes

ATOM subunits RNAi data:
Accession number: PXD027739
Username: reviewer_pxd027739@ebi.ac.uk
Password: 01P0YMT5

TbUbL1-myc IP data:
Accession number: PXD027652
Username: reviewer_pxd027652@ebi.ac.uk
Password: lBrA7YDo

ATOM69-RNAi +/- LMB data:
AAccession number: PXD031888
Username: reviewer_pxd031888@ebi.ac.uk
Password: GvwU4TSP

Field-specific reporting

Please select the one below that is the best fit for your research. If you are not sure, read the appropriate sections before making your selection.

- [x] Life sciences
- [ ] Behavioural & social sciences
- [ ] Ecological, evolutionary & environmental sciences

For a reference copy of the document with all sections, see nature.com/documents/nr-reporting-summary-flat.pdf

Life sciences study design

All studies must disclose on these points even when the disclosure is negative.

Sample size	The sample size was chosen based on the type of experiment and whether the chosen sample size gave reasonable reproducibility in several independent experiments. When data were consistent across the experiments, we considered the sample size to be sufficient.
Data exclusions	No complete optimised experiments were excluded
Replication	Each optimized experiment was reproduced at least three times with similar results.
Randomization	Experiments were not randomized because the cells used in the different experimental groups were derived from the same isogenic lines.
Blinding	-Determination of the percentage of the population showing release of TbUbL1-myc (Fig. 4a, 4b, 6a, 7a) was done on blinded datasets<br>-Quantification of the nuclear and cytosolic immunofluorescence intensities of the TbUbL1-myc (supplementary Fig. 6a, 6b) was done on blinded datasets<br>-For all other experiments the investigators were not blinded to group allocation during data collection or subsequent analysis. This approach is considered standard for biochemical experiments performed here also because phenotypic changes often are very obvious.

Reporting for specific materials, systems and methods

We require information from authors about some types of materials, experimental systems and methods used in many studies. Here, indicate whether each material, system or method listed is relevant to your study. If you are not sure if a list item applies to your research, read the appropriate section before selecting a response.

Materials & experimental systems

n/a	Involved in the study
☐	☒ Antibodies
☐	☒ Eukaryotic cell lines
☒	☐ Palaeontology and archaeology
☒	☐ Animals and other organisms
☒	☐ Human research participants
☒	☐ Clinical data
☒	☐ Dual use research of concern

Methods

n/a	Involved in the study
☒	☐ ChIP-seq
☒	☐ Flow cytometry
☒	☐ MRI-based neuroimaging

Antibodies

Antibodies used

The following polyclonal antibodies generated previously by our lab were used: VDAC (1), ATOM40 (2) and ATOM69 (2), COXIV (3), cytochrome c (4), TimRhomI (5), mtHsp60 (6), Alba3 (7). The polyclonal TbUbll rabbit antisera generated in this work was produced commercially by Eurogentec using peptide sequence CSEISGNHRSSEHNAG. The antibody was affinity purified against the antigen by Eurogentec.
Commercially available antibodies: mouse c-Myc (Invitrogen, clone 9E10, 13-2500), mouse HA (Biolegend, clone 16B12, 901515), rabbit anti-Ub (Proteintech, 10201-2-AP) and mouse EFla (Merck Millipore, clone CBP-KKl, 05-235). Secondary antibodies used were IRDye 680LT goat anti-mouse (LI-COR Biosciences, 925-68020), IRDye 800CW goat anti-rabbit (LI-COR Biosciences, 926-32211), goat anti-mouse Alexa Fluor 594 (ThermoFisher Scientific, A11032) and goat anti-rabbit Alexa Fluor 488 (ThermoFisher Scientific, A11070).

1) Pusnik, M. et al. The single mitochondrial porin of Trypanosoma brucei is the main metabolite transporter in the outer mitochondrial membrane. Mol. Biol. Evol. 26, 671-680 (2009).
2) Mani, J. et al. Mitochondrial protein import receptors in Kinetoplastids reveal convergent evolution over large phylogenetic distances. Nat Commun 6, 6646, doi:10.1038/ncomms7646 (2015).
3) Niemann, M. et al. Mitochondrial outer membrane proteome of Trypanosoma brucei reveals novel factors required to maintain mitochondrial morphology. Mol. Cell. Proteomics 12, 515-528 (2013)
4) Crausaz-Esseiva, A. et al. Temporal dissection of Bax-induced events leading to fission of the single mitochondrion in Trypanosoma brucei. EMBO Rep. 5, 268-273 (2004).
5) Harsman, A. et al. The non-canonical mitochondrial inner membrane presequence translocase of trypanosomatids contains two essential rhomboid-like proteins. Nat Commun 7, 13707, doi:10.1038/ncomms13707 (2016).
6) Chanez, A.-L., Hehl, A., Engstler, M. & Schneider, A. Ablation of the single dynamin of T. brucei blocks mitochondrial fission and endocytosis and leads to a precise cytokinesis arrest. J. Cell Sci. 119, 2968-2974 (2006).
7) Mani, J. et al. Alba-domain proteins of Trypanosoma brucei are cytoplasmic RNA-binding proteins that interact with the translation machinery. PLoS One 6, e22463, doi:10.1371/journal.pone.0022463 (2011)

Validation

This TbUbL1 antibody was validated using a T. brucei RNAi cell line targeting TbUbL1 which showed a band at the correct approximate MW which was not present when the RNAi was induced by the addition of tetracycline.
The validation for the other used antibodies is found in the following publications

1) Pusnik, M. et al. The single mitochondrial porin of Trypanosoma brucei is the main metabolite transporter in the outer mitochondrial membrane. Mol. Biol. Evol. 26, 671-680 (2009).
2) Mani, J. et al. Mitochondrial protein import receptors in Kinetoplastids reveal convergent evolution over large phylogenetic distances. Nat Commun 6, 6646, doi:10.1038/ncomms7646 (2015).

Eukaryotic cell lines

Policy information about cell lines

Cell line source(s)

All cell lines are based on the parental procyclic 29.13 Trypanosoma brucei cell line (1). This is a non commercial cell line. The production of transgenic derivatives of 29.13 is described in detail in the manuscript.

(1) Wirtz, E., Leal, S., Ochatt, C. & Cross, G. A. A tightly regulated inducible expression system for conditional gene knock-outs and dominant-negative genetics in Trypanosoma brucei. Mol. Biochem. Parasitol. 99, 89-101 (1999).

Authentication

Expression of tagged proteins: The expression of the tagged proteins has been verified using immunoblots (shown in the manuscript).
The different RNAi cell lines have been verified using Northern blots and/or immunoblots (see Supplementary Fig. 3).

Mycoplasma contamination	Trypanosomes are not a host for Mycoplasma
Commonly misidentified lines (See ICLAC register)	none
